# Editorial to “Can lead damage be ruled out using defibrillation threshold testing in patients with very high‐impedance shock leads?” Beyond the numbers: Reconsidering high shock impedance in ICD leads with DFT testing

**DOI:** 10.1002/joa3.70032

**Published:** 2025-03-04

**Authors:** Yoshinari Enomoto

**Affiliations:** ^1^ Department of Cardiology Center Hospital of the National Center for Global Health and Medicine Tokyo Japan

Implantable cardioverter defibrillators (ICDs) have been shown to reduce overall mortality in both the primary and secondary prevention of sudden cardiac death. However, lead failure remains the Achilles' heel of this therapy. Defibrillation threshold (DFT) testing was historically used to assess device function, including lead integrity, and to confirm device settings, such as sensing functionality. In recent years, however, DFT testing has become less commonly performed because of its procedural risks and limited impact on clinical outcomes.

In this issue of the *Journal of Arrhythmia*, Narita et al.^1^ provide valuable insights into the management of high shock impedance in transvenous ICD leads. Clinically, a shock impedance exceeding 200 Ω often raises concerns about lead failure, prompting consideration of lead replacement or additional lead implantation. However, this study revisits an older yet underutilized approach by employing DFT testing to evaluate true shock impedance (TSI). The authors demonstrate that DFT testing confirmed preserved lead functionality in patients with high shock impedance, thereby avoiding unnecessary lead replacement. Shock impedance in ICD leads can be measured using two methods: high‐voltage shock impedance (HVSI), which involves delivering a high‐energy shock, and low‐voltage subthreshold measurement (LVSM), which uses low‐energy pulses to approximate TSI. While HVSI is considered more accurate, its invasive nature and associated risks limit its application, particularly in high‐risk populations such as elderly patients or those with significant comorbidities. For patients with anatomical challenges, such as a persistent left superior vena cava (PLSVC), lead extraction or additional lead implantation carries significant procedural risks. By utilizing DFT testing to evaluate true shock impedance, Narita et al.^1^ propose a less invasive approach, which is particularly relevant in real‐world clinical scenarios where minimizing procedural risks is critical.

The frequency of ICD lead failure varies widely depending on lead type, patient characteristics, and follow‐up duration. According to previous reports, the annual electrical failure rate for non‐recalled ICD leads is approximately 0.6%.^2^ In contrast, certain recalled lead models have reported annual failure rates ranging from 2.6% to 4.8%.^3^ Moreover, a large longitudinal study reported that the 5‐year and 8‐year lead survival rates were 85% and 60%, respectively, with the annual failure rate increasing significantly over time, reaching as high as 20% per year after 10 years.^4^ These findings underscore the progressive nature of ICD lead failure and the importance of long‐term follow‐up. One of the key indicators of ICD lead failure is an increase in shock impedance. Regular in‐person device interrogation and remote monitoring are crucial for the timely detection of lead failure and can support informed clinical decision‐making.

The Endotak Reliance 0296 (Boston Scientific, Marlborough, MA, USA), the ICD lead evaluated in this study, is coated with GORE‐expanded polytetrafluoroethylene (ePTFE) to prevent tissue ingrowth around and between the shock coils. This lead is known to have the potential to develop calcification, which can result in elevated impedance and may interfere with defibrillation and pacing. Interestingly, high out‐of‐range impedance has been observed in this lead model without associated clinical adverse events.^1^ This suggests that elevated impedance may be a lead‐specific phenomenon. In such cases, DFT testing may serve as an effective tool to confirm lead functionality and prevent unnecessary interventions. This study highlights that, in select cases, DFT testing can still play a pivotal role in avoiding unnecessary lead replacement. This approach is particularly significant for elderly patients or those with complex anatomical conditions, where invasive procedures carry heightened risks.

As device technology continues to evolve, it is essential to recognize that the features and functions of ICD generators and shock leads vary across manufacturers. Refining the decision‐making process for ICD lead management, with careful consideration of lead‐specific characteristics and individual patient needs, will be crucial for optimizing outcomes and ensuring the long‐term success of ICD therapy.

## CONFLICT OF INTEREST STATEMENT

The author declares no conflicts of interest.

## FUNDING INFORMATION

This research did not receive any specific grants from funding agencies in the public, commercial, or not‐for‐profit sectors.
